# Electrical resistivity tomography combined with seismic data estimates heterogeneous distribution of near-seafloor concentrated gas hydrates within gas chimneys

**DOI:** 10.1038/s41598-024-65817-4

**Published:** 2024-07-01

**Authors:** Keiichi Ishizu, Ayako Oda, Tada-nori Goto, Takafumi Kasaya, Toshiki Watanabe, Hideaki Machiyama

**Affiliations:** 1 Graduate School of Science, University of Hyogo, 2167 Shosha, Himeji, Hyogo 671-2280 Japan; 2 Research Institute for Marine Resources Utilization, Japan Agency for Marine-Earth Science and Technology, 2-15 Natsushima, Yokosuka, Kanagawa 237-0061 Japan; 3 School of Science, University of Hyogo, 2167 Shosha, Himeji, Hyogo 671-2280 Japan; 4 Now at Weathernews Inc., Makuhari Techno Garden, 1-3 Nakase, Mihama, Chiba, Chiba 261-0023 Japan; 5 Earthquake and Volcano Research Center, Graduate School of Environmental Studies, Nagoya University, Furo-Cho, Chikusa-Ku, Nagoya, Aichi 464-8601 Japan

**Keywords:** Environmental sciences, Ocean sciences, Solid Earth sciences, Geophysics

## Abstract

Near-seafloor concentrated gas hydrates (GHs) containing large amounts of methane have been identified at various gas chimney sites. Although understanding the spatial distribution of GHs is fundamental for assessing their dissociation impact on aggravating global warming and resource potential, the spatial distribution of GHs within gas chimneys remains unclear. Here, we estimate the subseafloor distribution of GHs at a gas chimney site in the Japan Sea using marine electrical resistivity tomography data. The resulting two-dimensional subseafloor resistivity structure shows high anomalies (10–100 Ωm) within seismically inferred gas chimneys. As the resistivity anomalies are aligned with high amplitude seismic reflections and core positions recovering GHs, we interpret the resistivity anomalies are near-seafloor concentrated GH deposits. We also detect various distribution patterns of the high resistivity anomalies including 100-m wide and 40-m thick anomaly near the seafloor and 500-m wide anomaly buried 50 m below the seafloor, suggesting that GHs are heterogeneously distributed. Therefore, considering such heterogeneous GH distribution within gas chimneys is critical for in-depth assessments of GH environmental impacts and energy resources.

## Introduction

A gas hydrate (GH) is an ice-like compound containing abundant methane gas formed only under low-temperature and high-pressure conditions^1–4^. Natural GHs exist on or beneath the seafloor^5–9^ and in permafrost areas^10^. As GHs are rich in methane, their dissociation causes its release into the ocean and atmosphere, aggravating global warming^11–13^. In fact, methane emitted from GH dissociation may have contributed to global warming during the Paleocene–Eocene Thermal Maximum^14^. In contrast, GHs are also a potential energy resource^13^, and several offshore GH production tests have been conducted in Japan^15,16^ and China^17–19^. Thus, GHs are the subject of extensive research due to their negative environmental impact and potential as unconventional energy resources.

Submarine gas chimney sites are regions with high-flux gas inputs that are frequently accompanied by GHs^20^. GHs have been identified at gas chimney sites within the Japan Sea^21^, offshore California^22^, offshore Sakhalin Island^23^, South China Sea^24^, and offshore Norway^25^. They occur as massive aggregates at high concentrations and are mostly located near the seafloor^7,20^. The dissociation of near-seafloor concentrated GHs within gas chimneys may impact the environment^26^. Methane emitted at the seafloor from the dissociation is partially consumed by aerobic microbial oxidation in the water column while the remainder can reach the ocean–atmosphere interface due to the overlying shallow water depth at high latitudes^13,26^. Although the dissociation of near-seafloor GHs at mid and low latitudes is considered less of an issue due to the thick water column^13^, GH chunks released from the seafloor by their collapse^21^ may impact the environment even at mid and low latitudes by reaching the ocean–atmosphere interface. Water lowering, temperature change, submarine landslides, and earthquakes can trigger GH collapse^27^. As energy resources, near-seafloor concentrated GHs are currently less attractive than those in sand layers as production technology for conventional oil and gas is unsuitable for near-seafloor GHs. However, owing to their high concentration and abundance, future development of near-seafloor GHs at gas chimney sites has become a particular area of interest in Japan^20,28^.

Understanding the spatial distribution of GHs is fundamental for assessing the impact of their dissociation on climate and resource potential. Although drilling surveys are crucial for investigating GH distributions, the sparsity of drilling points results in low horizontal spatial resolution (e.g., 1 km spacing for Ocean Drilling Program Leg 204^5^). Meanwhile, geophysical surveys are useful for investigating the spatial distribution of GHs^29,30^. Seismic surveys provide the structural distribution and the geological background relevant to GHs. These surveys also detect GH and gas-related features such as bottom simulating reflectors, high-amplitude seismic reflections, and seismic blanking zones^29,31,32^. However, relying solely on seismic surveys is insufficient for determining GH and gas saturation because even a small percentage of GH or gas can lead to high-amplitude seismic reflections and seismic blanking zones^33^. As resistivities are sensitive to GH and gas saturation, electrical and electromagnetic surveys investigating subseafloor resistivity structures have been integrated with seismic surveys for a more robust estimation of GH distribution and its saturation^34,35^. GH-bearing sediments show higher resistivity values (generally 2–1000 Ωm) than the surrounding sediments and higher resistivity values correlate with greater GH saturation^20,36–44^.

Electrical and electromagnetic surveys have determined the resistivity distribution of GH at submarine gas chimney sites. Constable et al.^45^ applied a controlled source electromagnetic (CSEM) survey using a towed system with four receivers (150–450 m offsets from a transmitter dipole) in the San Diego Trough, offshore southern California. Meanwhile, Schwalenberg et al.^34^ used a seafloor-towed CSEM system with four receivers (160–750 m offset from a transmitter dipole) at Opouawe Bank, Hikurangi Margin. They identified potential GHs as high-resistivity anomalies and found that the upper limit of the highest GH saturation zone occurs at 50–150 mbsf^34,45^. In contrast, the highest GH saturation zone occurs on the seafloor or immediately below the seafloor (< 50 mbsf) at various gas chimney sites^5,20,46^ including the eastern margin of the Japan Sea. Compared to GHs with the highest saturation at 50–150 mbsf, GHs with the highest saturation near the seafloor can exert stronger influences on the environment; however, their spatial distribution remains unclear.

**Figure 1:**
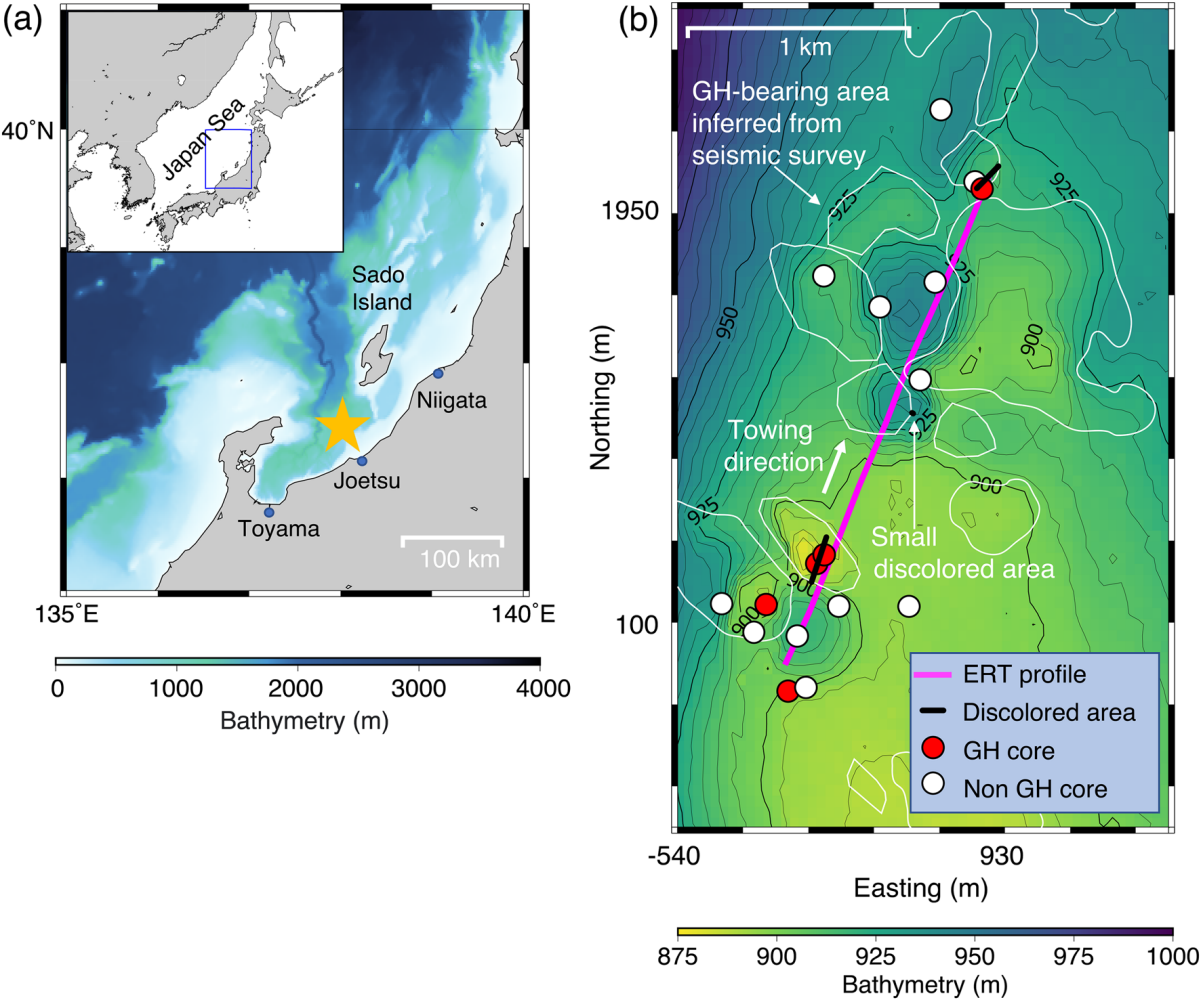
Map of the study area. (**a**) Location of Umitaka Spur, the eastern margin of the Japan Sea, is indicated by a yellow star. (**b**) Event map of Umitaka Spur. The magenta line represents a survey profile with a towed electrical resistivity tomography (ERT) system^38^. Black lines indicate discolored seafloor areas, detected based on video observations along a towing profile^38^ conducted prior to the ERT survey. The discolored areas comprise microbial mats, carbonate, and gas hydrate (GH). Red and white circles denote the location of piston coring with and without GH samples, respectively^38^. White lines show outlines of GH-bearing areas inferred from seismic surveys; the areas were determined by amplitude anomalies in three-dimensional seismic reflection data and seafloor topography^53^. We generated the maps in (**a**,**b**) using the Generic Mapping Tools 5.4.5 (https://www.generic-mapping-tools.org/).

**Figure 2:**
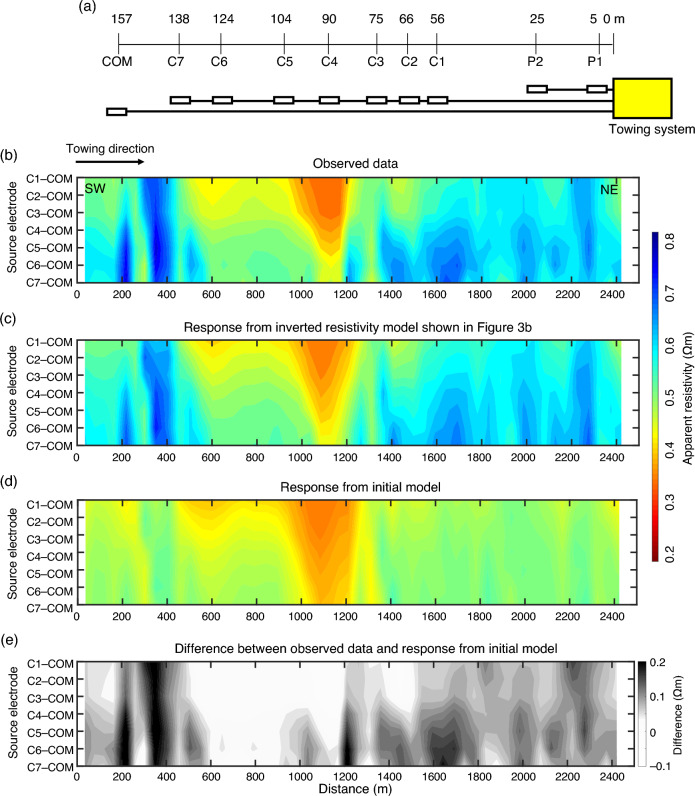
Electrode configuration of the electrical resistivity tomography (ERT) system, observed ERT data, and model responses. (**a**) Electrode location of the ERT system^38^. The ERT system uses eight current electrodes, C1–C7 and COM, and two receiver electrodes, P1 and P2. Current was transmitted using electrode pairs: C1–COM, C2–COM, C3–COM, C4–COM, C5–COM, C6–COM, and C7–COM. (**b**) Observed apparent resistivity data^38^. (**c**) Response from the inverted resistivity model shown in Fig. 3b. (**d**) Response from an initial model comprising a 1.0 Ωm homogeneous subseafloor layer and a 0.35 Ωm seawater layer. (**e**) Difference between the observed data and the initial model response. (**d**) and (**e**) highlight data that the homogeneous sub-seafloor structure of 1 Ωm cannot explain. The vertical axis is based on source electrode pairs. The C7–COM pair reflects deeper resistivity structures than C1–COM because of longer source–receiver septation. Data are shown with southwest on the left (start point of towing) and northeast on the right (end point of towing). The observed apparent resistivity at a horizontal distance of 900–1200 m is low because of the high tow height of the system.

We use marine electrical resistivity tomography (ERT) data obtained from Umitaka Spur on the eastern margin of the Japan Sea (Fig. 1) to estimate distribution of near-seafloor concentrated GHs at a gas chimney site. Piston coring and drilling have confirmed near-seafloor concentrated GHs in Umitaka Spur^21,38,47^. The ERT data were obtained in 2005 in an ERT survey conducted by Goto et al.^38^ using ten deep-towed electrodes for transmitters and receivers attached to a 160-m towing cable (Fig. 2a). The short-spacing electrode array with the near-seafloor-towed ERT system enables resistivity imaging up to 100 mbsf. The ERT data showed that the high apparent resistivity zone corresponded to the GH zone^38^. However, the subseafloor resistivity structure was not estimated due to the lack of an available inversion code. We apply our recently developed two-dimensional (2D) inversion code^48^ to the ERT data and obtain a subseafloor resistivity structure in Umitaka Spur. To estimate GH saturation, we incorporate seismic data and piston core results into the interpretation of our resistivity model.

The Japan Sea located in the northern part of the western Pacific marginal basin is a back-arc basin formed by continental rifting of the eastern margin of Eurasia^49^. Extensions of the Japan Sea occurred 32–10 Ma, while the major opening and volcanism in the Japan Sea were completed before 18 Ma^49^. Tectonic inversion from extension to compression occurred at approximately a few Ma^50,51^, causing various normal faults to be reactivated as reverse faults^50,51^. Owing to the formation of the Japan Sea, structures such as faults, folds, and sediment layers rich in organic matter developed on its eastern margin^21,51^. Folds and faults form effective traps for hydrocarbon storage and act as conduits for the migration of deep gases to the seafloor^21^, which are essential for near-seafloor GH evolution.

Near-seafloor concentrated GHs have been identified within gas chimneys on the eastern margin of the Japan Sea. Accordingly, this area has been investigated for the presence of GHs^21,28,38,52–56^. Multi-beam echo sounder and sub-bottom profiler surveys have detected 1742 gas chimneys as acoustic blanking zones along the eastern margin of the Japan Sea and around Hokkaido^47^, indicating an abundance of GHs. Owing to the abundance of GHs and the availability of scientific data on the eastern margin of the Japan Sea, this area has been deemed important for conducting GH studies.

Umitaka Spur, with a sea depth of 850–1000 m, is located 70 km southwest of Sado Island and is the most studied GH area on the eastern margin of the Japan Sea (Fig. 1). Near-seafloor concentrated GHs have been confirmed by piston coring and drilling in this area^21,38,47^, and seismic and acoustic surveys have detected gas chimneys aligned in the NNE–SSW direction; gas chimneys carry methane gas from deep-seated sources to the seafloor^21,54^. Gases extracted from the hydrates were composed of variable mixtures of thermogenic and microbial methane, with δ^13^C of methane ranging from –32‰ to –86‰^20^. Moreover, mounds and pockmarks have been observed on the seafloor and their formation has been related to the occurrence and collapse of GHs^21,57^. Pockmarks with several hundred meters in diameter have resulted from large GH collapses triggered by falling sea levels during the last glacial maximum period (12–24 kyrBP)^21,58^. The 120 m decrease in the Japan Sea level during this period shallowed the depth of the base of GH stability (BGHS) by 20 m, causing dissociation of GHs located 20 m above the prior BGHS depth^21^. The released methane gas rose, causing substantial GH accumulation near the seafloor due to the reaction between methane gas and water^21^. Consequently, GH mounds became unstable due to the high buoyancy of GHs in seawater, and triggered self-collapse, forming pockmark structures several hundred meters in diameter^21^.

The resistivity structure obtained from the inversion of the ERT data specified 10–100 Ωm high-resistivity anomalies within seismically inferred gas chimneys. As the resistivity anomalies were aligned with high amplitude seismic reflections and core positions recovering GHs, we interpreted that the resistivity anomalies are near-seafloor concentrated GH deposits. The high resistivity anomalies showed various distribution patterns within the gas chimneys including 100-m wide and 40-m thick anomaly near the seafloor and 500-m wide anomaly buried 50 m below the seafloor. This indicates that GHs and focused gas are heterogeneously distributed within gas chimneys.

## Results

**Figure 3:**
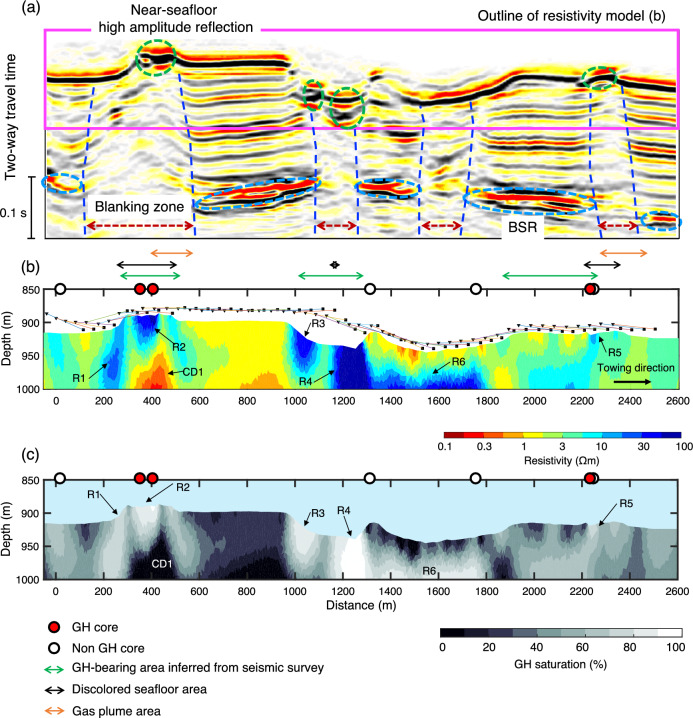
Seismic section, inverted resistivity model, and subseafloor saturation rates. (**a**) Seismic section below the electrical resistivity tomography (ERT) towing profile (Fig. 1b). The seismic section is extracted from three-dimensional seismic survey data^59^. Two-way travel time of 0.1 s corresponds to a depth of 85–90 m for velocities of 1700–1800 m/s^59^. A magenta rectangular represents an outline of the resistivity model in (**b**). Green and light blue dashed circles show near-seafloor high amplitude reflections and bottom simulating reflectors (BSRs), respectively. Near-vertical, dark-blue dashed curves indicate the boundaries of seismic blanking zones. (**b**) Vertical cross-section of the inverted resistivity model below the towing profile. R1–R6 and CD1 are high (> 10 Ωm) and low (< 1.0 Ωm) resistivity zones, respectively. (**c**) Distribution of gas hydrate (GH)/gas saturation rates. Saturation rates are derived from the subseafloor resistivity values in (**b**) using Archie’s law. Small black squares and triangles indicate the locations of the head and tail of the towing system of ERT data. Each location of the head and tail is connected by lines. Red and white circles indicate the positions of piston cores with and without GH, respectively, within 150 m of the ERT towing profile^38^. Green arrows show GH areas inferred from seismic surveys^53^ (Fig. 1b). Black arrows show discolored seafloor areas detected based on video observations^38^. Orange arrows show methane plumes zones^21^.

The ERT data were obtained from Umitaka Spur along a survey line of approximately 2.5 km^38^ (see Method section; Fig. 1b). Figure 2b shows a pseudo-section of the apparent resistivity of observed ERT data, reflecting the seawater and subseafloor resistivity distribution below the survey line. The apparent resistivity also depends on the towing height. Given that the non-GH background resistivity value is 1 Ωm^38^, differences between the observed apparent resistivity data and the 1 Ωm model response provide information on subseafloor high resistivity zones mitigating the effects of the towing height (Fig. 2d,e). Positive differences > 0 Ωm, indicating the existence of high-resistivity anomalies, were observed at a horizontal distance (200–600 m, 900–1200 m and 1200–2300 m). Although a low apparent resistivity zone was observed at a horizontal distance of 900–1200 m, positive differences were observed for source electrodes of C5–COM, C6–COM, and C7–COM in this zone. Therefore, this zone was strongly influenced by seawater owing to the large distance (> 30 m) between the towing system and the seafloor; the low apparent resistivity values do not necessarily indicate the existence of low-resistivity anomalies below the seafloor. Positive differences were observed in seismic blanking zones (Fig. 3a), indicating that high resistivity zones may exist in the gas chimney structures. Inversion of the observed ERT data facilitates the incorporation of seawater and towing height effects and quantitatively determines the resistivity structure below the seafloor.

The inversion model (Fig. 3b) revealed the prominent high-resistivity zones of R1–R6 (> 10 Ωm) with various distribution patterns. The recovery of the high-resistivity zones R1–R6, can be explained by positive differences between the observed data and the 1 Ωm seafloor model response (Fig. 2e). Seismic blanking zones were observed at and below R1–R6, indicating R1–R6 are related to the gas chimney structures (Fig. 3a), whereas non-GH background resistivity zones (~ 1 Ωm) was in the non-gas chimney regions (regions without seismic blanking). R1 to R5 were located near the seafloor, whereas R6 was buried at 50 mbsf. The near-seafloor resistors R1–R5 differed in their horizontal reach and vertical thickness. For example, R1 was 70 m wide and 100 m thick, whereas R2 was 100 m wide and 40 m thick. R1 and R4 extended to the deeper subseafloor regions, whereas R2, R3, and R5 extended to tens of meters below the seafloor. R2 and R5 coincided with the positions of the piston cores, which recovered GHs^38^. R1, R2, and R5 were on the mound structure, whereas R3 and R4 were on the pockmark structure; R1–R5 were consistent with GH-bearing areas inferred from seismic surveys^53^. Methane plumes were observed at R2 and R5^21^. R2–R5 were located at zones of near-seafloor high amplitude reflections in the seismic section (Fig. 3a,b). Note that the three-dimensional (3D) seismic survey was aimed at deep reservoirs at 4000–5000 mbsf and was not intended for shallow GH; the wavelength was ~ 50 m for 30–40 Hz center frequency and 1800 m/s velocity^59^.

Subseafloor GH/gas saturation in pore spaces (*S*_*g*_) was calculated from the resistivity model using Archie’s equation: *a* = 1.0, *n* = 2.0, and *m* = 2.5 (see Method section; Fig. 3c); the porosity values are described in Eq. (6). High saturation rates > 60% were estimated in the high-resistivity zones of R1–R6 (Fig. 3c). The high GH saturation rates for R1–R6 were also computed with different *m* = 2.2–2.8. *S*_*g*_ of 100 Ωm (R2) calculated with *m* = 2.2, 2.5, and 2.8 were 91.4%, 91.0%, and 90.5%, respectively.* S*_*g*_ of 13 Ωm (R5) calculated with *m* = 2.2, 2.5, and 2.8 were 76.2%, 75.0%, and 73.7%, respectively. These results indicated that the *S*_*g*_ estimation of R1–R6 only differed by a maximum of 3% with an *m* = 2.2–2.8 and the influence of the cementation factor on the saturation estimate decreased when the formation resistivity or saturation was higher.

## Discussion

**Figure 4:**
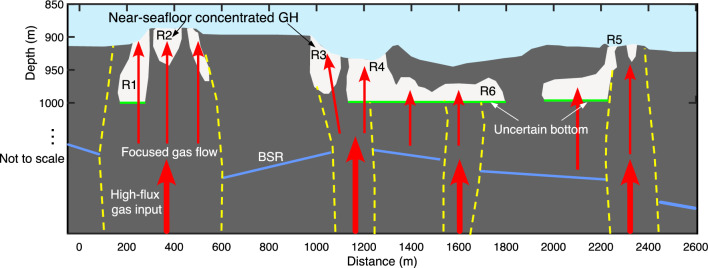
Schematic diagram of the gas hydrate (GH) evolution model at a gas chimney site in the Japan Sea. The white zones are areas with peak GH/gas saturation > 60% and red arrows represent focused gas pathways. Light green lines indicate uncertain bottoms of the GHs that are unconstrained by our electrical resistivity tomography data. Blue lines and yellow dashed curves represent bottom simulating reflectors (BSRs) and outlines of seismically inferred gas chimneys. The figure is not to scale.

Our resistivity model reveals near-seafloor high-resistivity zones of R1–R5, interpreted as near-seafloor concentrated GHs. The obtained resistivity values for R1–R5 (10–100 Ωm) are consistent with those reported for GHs at other gas chimney sites^34,45^. R1–R5 are located on seafloor mounds and pockmarks; the distribution of R1–R5 is consistent with GH-bearing areas inferred from seismic surveys^53^. R1, R2, R4, and R5 overlap with discolored areas detected based on seafloor video observations^38^; the discolored areas consist of microbial mats, carbonate, and GH. R2 and R5 coincide with the positions where GHs were identified by piston cores. The piston cores collected massive-type GH samples at R2^38^. The piston core at R5 recovered the soupy mud, implying the dissociation of GH during piston core recovery^38^. The higher resistivity values of R2 than R5 are consistent with the core recovery of GHs. High amplitude reflections observed at R2–R5, suggest that R2–R5 are near-seafloor concentrated GHs. High amplitude reflections are not observed at R1, implying that GH saturation rates of R1 are lower than those of R2–R5. Seismic blanking zones below R1–R5 imply high-flux gas upwells to R1–R5. Although resistivity alone cannot discriminate GH or gas^34^, our resistivity model combined with preceding seismic data and core recovery observations reinforces our interpretation that GH is the predominant constituent for the high-resistivity zones of R2–R5, with high-flux gas inputs resulting in GH accumulation at R1–R5 (Fig. 4).

High GH/gas saturations exceeding 60% in R1–R4 indicate the presence of high saturation GH deposits with substantial volumes. These deposits exhibit horizontal dimensions ranging from 50 to 150 m and vertical dimensions spanning 40 m to 80 m. In the Joetsu Basin, which encompasses our study area, coring was performed at nine drill sites within seismically inferred gas chimneys and core analysis reported that the average volume fraction of GHs at each drill site ranges from 35% to 86% of the sedimentary sequences^20^. Comparing these values with our GH saturation model suggests that our GH saturation model provides reasonable estimates.

Horizontally elongated R6, buried at a depth of 50 mbsf, differs geometrically from the near-seafloor GHs of R1–R5. Such horizontally elongated GHs have been found above BSRs in various GH areas. In facts, BSRs are observed below R6 at a horizontal distance of 1300–1500 m (Fig. 3a). Our ERT data cannot constrain the bottom of R6 due to the shallow exploration depth; however, the bottom of the GH-bearing zone of R6 is constrained by the BGHS depth or BSRs. The BGHS depth in this area is reportedly 115 mbsf^21^. The thickness of the GH-bearing zone of R6 will be 70 m if it extends to the BGHS depth, corresponding to the upper-bound estimate of the GH-bearing zone of R6. Moreover, given that high amplitude reflections are not observed at R6, GH saturation rates of R6 are lower than those of R2–R5, while the free gas saturation rates of R6 are higher than those of R2–R5.

The GH/gas saturation rates of R2 and R3 are the highest near the seafloor and decrease with depth. A thermodynamic equilibrium model, which considers a three-phase zone, can explain much higher GH saturation near the seafloor than in the deep parts of a gas chimney^4,60,61^. That is, free gas upwelling from depths enters the GH stability zone, where the reaction of free gas with seawater forms GH; porewater salinity increases as a result of GH formation; increasing salinity brings the local thermodynamic condition closer to a three-phase equilibrium of seawater, GH, and free gas. GH formation continues until the system reaches a three-phase equilibrium due to the sufficiently elevated salinity. The free gas can rise to the seafloor after the GH stability zone reaches the three-phase equilibrium. In the case of high gas flux with no water flux in sand, high salinity, low water content, and high GH saturation are required to maintain the three-phase equilibrium conditions near the seafloor^61^. In contrast, low salinity, high water content, and low GH saturation explain the three-phase equilibrium conditions in deeper parts, including immediately above the BGHS^61^. These numerical predictions are consistent with the GH saturations of R2 and R3 observed in this study. The conductor CD1 below R2 exhibits unusually high conductivities within the GH stability zone, suggesting that it might be an artifact resulting from the inversion. As CD1 is situated in a circular GH area inferred from seismic surveys^53^ (Figs. 1b and 3b), 3D effects of the circular structure to our 2D inversion could lead to the artifact.

Our resistivity model estimates potential GH zones within the seismically inferred gas chimneys by combining the seismic data, the piston cores, and the numerical thermodynamic model. The estimated GH saturation rate for R2 and R3 is the highest near the seafloor and decreases with depth; this is explained well by the thermodynamic equilibrium model^60^. Various distribution patterns of potential GH deposits are found within the seismically inferred gas chimneys: (1) GHs extending from the seafloor to deeper zones (R1 and R4), (2) GHs extending from the seafloor to several tens of meters below the seafloor (R2, R3, and R5), and (3) horizontally elongated GH buried at a depth of 50 mbsf (R6). This indicates that GHs and focused gas flows are heterogeneously distributed within gas chimneys. Such a variety of GH distributions were not considered when determining their dissociation impact on environments. Incorporating the near-seafloor GH distribution into analyses of their dissociation impact on environments will improve the assessment accuracy. Moreover, considering the various GH distributions will improve the accuracy of GH resource estimations.

## Methods

### Deep-towed marine ERT method

Deep-towed marine ERT methods used electric current sources and receivers to investigate subseafloor resistivity structures^38,48,62^. The ERT data used in this study were acquired by Goto et al.^38^ during the research cruise KY05–08 in August 2005. The ERT system comprised eight source and two receiver electrodes attached to a 160-m long floating cable (Fig. 2a) that was behind the main system, including cameras, conductivity–temperature–depth (CTD) sensors, an altimeter, and an alternating current transformer^38^. The ERT system was towed by a ship at an altitude of less than a few tens of meters from the seafloor with a towing speed of ~ 1 knot (0.5 m/s). These short-spacing electrode arrays with the near-seafloor-towed ERT system enabled near-seafloor resistivity imaging. The ERT system used a source signal comprising sinusoidal with a period of 4 s; a source signal was sequentially sent to the seven dipoles for one period at a constant voltage of 72 V peak to peak. Accordingly, one source signal sequence took 28 s. The ERT system recorded the electrical potential at the P1 and P2 electrodes. The sampling rate of source current and receiver potential data was 0.2 s. Apparent resistivity was computed by stacking data for three sequences, i.e., 84 s, corresponding to ~ 50 m array movement. To mitigate the effects of the metallic cable and deep-tow frame of the ERT system, the apparent resistivity data were calibrated by conducting measurements in the water column at a depth of 400 m. The calibration factors of the apparent resistivity were 1.05, 1.03, 1.02, 1.14, 1.13, 1.10, and 1.08 for source dipoles of C1–COM, C2–COM, C3–COM, C4–COM, C5–COM, C6–COM, and C7–COM, respectively. An acoustic supershort-baseline (SSBL) navigation system provided positioning information on the transponders attached to the deep-tow frame and the cable tail. Goto et al.^38^ provide further details on the ERT system.

The apparent resistivity formulas help interpret ERT data features^38,48,62^. The apparent resistivity was determined from the observed voltage, source current, and geometries of the source and receiver electrodes using Eq. (1):

1 $${\rho }_{a}=4\pi \frac{V}{I}\left(\frac{1}{{r}_{{c}_{1}{p}_{1}}}-\frac{1}{{r}_{{c}_{1}{p}_{2}}}-\frac{1}{{r}_{{c}_{2}{p}_{1}}}+\frac{1}{{r}_{{c}_{2}{p}_{2}}}\right)$$

where ${\rho }_{a}$ is apparent resistivity (Ωm), *V* is receiver voltage (V), *I* is source current (A), and *r* is the distance between electrodes (m). Subscripts *c* and *p* indicate the current and receiver electrodes, respectively; ${\rho }_{a}$ represents the resistivity, assuming a homogeneous resistivity structure.

### Inversion of observed ERT data

The ERT inversion estimated the subseafloor resistivity structure from the apparent resistivity data. We used the 2D inversion code developed by Ishizu et al.^48^ to obtain the 2D resistivity structure below the towing profile. The inversion minimizes the following objective function *U*,

2 $$U={\left(\mathbf{d}-\mathbf{F}\left[\mathbf{m}\right]\right)}^{\text{T}}{\mathbf{C}}_{\mathbf{d}}^{-1}\left(\mathbf{d}-\mathbf{F}\left[\mathbf{m}\right]\right)+\mu {\left({\mathbf{m}-\mathbf{m}}_{0}\right)}^{\text{T}}{\mathbf{C}}_{\mathbf{m}}^{-1}\left({\mathbf{m}-\mathbf{m}}_{0}\right)$$

where **m** is a model vector,** m**_0_ is a prior model vector, **d** is the observed data vector, **F**[**m**] represents the forward modeling response, **C**_**m**_ represents a model covariance matrix,** C**_**d**_ is a data covariance matrix, and $\mu$ is a tradeoff parameter between data misfit and model roughness. The first derivative roughness penalty was used for ${\mathbf{C}}_{\mathbf{m}}^{-1}$. Owing to the non-linearity of the ERT inversion, the inversion applied an iterative approach based on the Gauss–Newton algorithm. Inversion with the Gauss–Newton algorithm generally converges after a small iteration number^63^. The tradeoff parameter $\mu$ was determined by an L-curve criterion^64^. The root mean square (RMS) data misfit was defined to measure overall data fitting per Eq. (3):

3 $$\text{RMS misfit}=\sqrt{\frac{{\left(\mathbf{d}-\mathbf{F}\left[\mathbf{m}\right]\right)}^{\text{T}}{\mathbf{C}}_{\mathbf{d}}^{-1}\left(\mathbf{d}-\mathbf{F}\left[\mathbf{m}\right]\right)}{N}}$$

where *N* is the number of data. The forward modeling used the finite element method with unstructured triangular meshes because the unstructured triangular meshes can model the seafloor topography efficiently^48^. The total number of apparent resistivity values input into the inversion code (*N*) was 357. The initial and prior models for the inversion consisted of 1.0 Ωm homogeneous subseafloor and 0.35 Ωm seawater layers. This sea resistivity value was the average value of the seawater resistivity measured using the CTD sensor during the ERT survey^38^. The mesh number for forward modeling was 20,318. For accurate computation, each mesh size was set to < 30 m^2^ in areas with high data sensitivity (i.e., a horizontal distance of −100 to 2600 m and a depth of from 800 to 1040 m). The resistivity of the sea layer was fixed at 0.35 Ωm during the inversion, which only updated the subseafloor region. Consequently, the total number of unknown model parameters was 9935. A minimum error setting of 2% was used for inversion. Seafloor topography was modeled as the sum of the CTD depth and altitude from the seafloor, measured using an altimeter. The altimeter data were missing for the seafloor topography at a horizontal distance of 1000–1250 m; thus, the topography of this section was supplemented with 50 m × 50 m bathymetric data (Fig. 1b).

The 2D inversion, with a fixed tradeoff parameter $\mu$ = 30, yielded a resistivity model below the seafloor after three iterations (Fig. 3b), and the RMS data misfit was 1.76. A tradeoff parameter of 30 was selected as the curvature of the L-curve was the largest for the parameter (Supplementary Fig. 1). The model explains the major features of the observed data (Fig. 2b,c; Supplementary Fig. 2). Meanwhile, the data fit worsened at 250–300 m and 1200–1250 m due to possible 3D topography effects.

### Error of tail angle and towing altitude

The ERT data included the systematic errors of the tail angle and towing altitude, which can distort inversion results. The standard error of the tail depth monitored by the SSBL system was ~ 0.5 m^38^; thus, the tail angle error was < 0.2°. A synthetic model study demonstrated that a tail error up to 1° did not strongly influence the inversion result of 2D ERT data^65^. Thus, we concluded that the systematic error of the tail angle did not significantly impact the inverted model.

The towing altitudes were measured using ultrasonic reflections from the seafloor^38^ and may have been underestimated as complex structures, such as mounds, can cause wave reflections from the side rather than directly below the towing system. Moreover, altitude data were missing at horizontal distance of 1000–1250 m and the altitudes may have been largely overestimated or underestimated. A synthetic model study reported that if the towing height was underestimated by 2.5 m (e.g., recorded height was 7.5 m, the actual towing height was 10 m), the inverted resistivity model did not differ significantly^65^. The error of the recorded altitude data was considered to be < 2.5 m. Although R3 and R4 were located on the altitude missing positions, the seismic section specified near-seafloor high amplitude reflections at R3 and R4 positions; this consistency supports the credibility of the resistivity model.

### Estimation of GH/gas saturation

The GH/gas saturation rates were estimated from the subseafloor resistivity values using Archie’s law—an empirical law that describes the relationship between electrical resistivity and pore spaces in sedimentary rocks^66^. Saturation estimates obtained from resistivity data using Archie’s law have been used in various studies^34–36,67^. Archie’s law is expressed by Eq. (4):

4 $${S}_{w}={\left(\frac{a{R}_{w}}{{\phi }^{m}{R}_{t}}\right)}^\frac{1}{n}$$

where *S*_*w*_ is water saturation rate in pore spaces, *R*_*w*_ is the resistivity of pore water (Ωm) set to the seawater resistivity of 0.35 Ωm, *R*_*t*_ represents bulk resistivity (Ωm), $\phi$ is the porosity of sediment, *a* is the tortuosity factor, *m* is the cementation factor, and *n* is the saturation exponent^36,66^. Assuming that GH and/or gas fills the remaining pore space, the GH/gas saturation rate in the pore spaces (*S*_*g*_) is defined by Eq. (5):

5 $${S}_{g}={1}-{S}_{w}$$

The tortuosity factor (*a*) and saturation exponent (*n*) were selected as 1.0 and 2.0, respectively, according to a study by Schwalenberg et al.^34^; a cementation factor (*m*) ranging from 2.2 to 2.8 has been used to estimate saturation in drilled GH areas^34^. Thus, given that the information required to determine the value of *m* was unavailable for this study, we applied the *m* = 2.2–2.8 range. Goto et al.^68^ reported a vertical porosity function for the Joetsu Basin, including our study area Umitaka Spur, using Eq. (6):

6 $$\phi (z) \, =0.718\exp(-1.07z\times {10}^{-3})$$

where *z* is the depth below the seafloor (m); this function was derived from core samples obtained from a depth up to 3081 mbsf. The closest drilling site (MD179-3304) was within 1 km of the ERT profile, and the geological setting of the drill sites was comparable to our survey area. We applied Eq. (6) to the porosity values used in Archie’s law to obtain *S*_*w*_ and *S*_*g*_.

### Supplementary Information

Supplementary Figures.

## Data Availability

The inverted resistivity and estimated GH saturation models are available on the Zenodo open-access repository operated by the CERN: 10.5281/zenodo.12526329. The ERT data are available from the corresponding author; however, there is a possibility of limited use of the ERT data because of political reasons concerning the Japanese government. The seismic data used in Fig. 3a can be requested to Japan Organization for Metals and Energy Security.
