# Supplementary material for: Electrical resistivity tomography combined with seismic data estimates heterogeneous distribution of near-seafloor concentrated gas hydrates within gas chimneys
Source: Sci Rep. 2024 Jul 1;14:15045. doi: 10.1038/s41598-024-65817-4 (PMC11217437; doi:10.1038/s41598-024-65817-4)
Supplement: Supplementary file 1 — Supplementary Figures. [file 41598_2024_65817_MOESM1_ESM.docx]

**Supplementary Information**

**Electrical resistivity tomography combined with seismic data estimates heterogeneous distribution of near-seafloor concentrated gas hydrates within gas chimneys**

Keiichi Ishizu^1,2^, Ayako Oda^3,4^, Tada-nori Goto^1,2^, Takafumi Kasaya^2^, Toshiki Watanabe^5^, Hideaki Machiyama^2^

^1^Graduate School of Science, University of Hyogo, 2167 Shosha, Himeji, Hyogo 671-2280, Japan

^2^Research Institute for Marine Resources Utilization, Japan Agency for Marine-Earth Science and Technology, 2-15 Natsushima, Yokosuka, Kanagawa 237-0061, Japan

^3^School of Science, University of Hyogo, 2167 Shosha, Himeji, Hyogo 671-2280, Japan

^4^Now at Weathernews Inc., Makuhari Techno Garden, 1-3 Nakase, Mihama, Chiba, Chiba 261-0023, Japan

^5^Earthquake and Volcano Research Center, Graduate School of Environmental Studies, Nagoya University, Furo-cho, Chikusa-ku, Nagoya, Aichi, 464-8601, Japan

Corresponding author: Keiichi Ishizu

Email: k.ishizu@sci.u-hyogo.ac.jp; Phone: +81-79-267-4942

ORCID: https://orcid.org/0000-0002-3663-182X

|  |
| --- |

**Supplementary Figure 1. Root mean square (RMS) data misfit and roughness of inverted models with different values of tradeoff parameter (*μ*)**

|  |
| --- |

**Supplementary Figure 2. Electrical resistivity tomography data fitting.** Black points with error bars and red lines represent observed apparent resistivity data and the response obtained from the inversion model, respectively. From top to bottom, the results are shown for **(a)** C1–COM, **(b)** C2–COM, **(c)** C3–COM, **(d)** C4–COM, **(e)** C5–COM, **(f)** C6–COM, and **(g)** C7–COM as source electrodes. Error bars are smaller in (a) than in (g) owing to the longer dipole length of C1–COM than of C7–COM.
